# Mucosa and microbiota – the role of intrinsic parameters on intestinal wound healing

**DOI:** 10.3389/fsurg.2022.905049

**Published:** 2022-07-22

**Authors:** Matthias Kelm, Friedrich Anger

**Affiliations:** Department of General, Visceral, Transplant, Vascular and Pediatric Surgery, University Hospital of Wuerzburg, Wuerzburg, Germany

**Keywords:** epithelial cells, mucosal healing (MH), microbiota, inflammatory bowel disease, anastomotic leakage

## Abstract

Mucosal healing in the gut is an essential process when it comes to chronic inflammatory disorders such as inflammatory bowel diseases (IBD) but also to the creation of intestinal anastomosis. Despite an improvement of surgical techniques, the rates of anastomotic leakage remain substantial and represent a significant health-care and socio-economic burden. Recent research has focused on intrinsic factors such as mucosal linings and differences in the intestinal microbiota and identified specific endoluminal bacteria and epithelial proteins which influence intestinal wound healing and re-establishment of mucosal homeostasis. Despite the lack of large clinical studies, previous data indicate that the identified bacteria such as aerotolerant lactobacilli or wound-associated Akkermansia muciniphila as well as epithelial-expressed sialyl Lewis glycans or CD47 might be critical for wound and anastomotic healing in the gut, thus, providing a potential novel approach for future treatment strategies in colorectal surgery and IBD therapy. Since microbiota and mucosa are interacting closely, we outline the current discoveries about both subsets in this review together to demonstrate the significant interplay

## Introduction

Impaired intestinal mucosal healing is a hallmark of inflammatory bowel diseases (IBD) and anastomotic leakage. Current IBD treatment mainly focuses on the immune system but the numbers of patients who still require surgery or suffer from side effects remain high (1). Since the incidence of IBD is increasing worldwide and numbers of anastomotic leakage remain significant (2, 3), insufficient mucosal healing represents a major socioeconomic burden making the development of novel therapeutic approaches urgently necessary to improve patient care. While endoscopic assessment of mucosal healing represents an established parameter to evaluate disease development (4, 5), specific treatment strategies to enhance wound healing are lacking. Therefore, an improved understanding of interactions between various gut-specific factors on wound healing and reconstitution of mucosal homeostasis is crucial.

The selectively and dynamically permeable barrier between luminal components and the basolateral membrane is established and maintained by the epithelium consisting of enterocytes and goblet cells. Intercellular connections between the epithelial cells are formed by intercellular junctions, namely the tight junctions, adherence junctions, desmosomes and gap junctions (6, 7). The goblet cells cover the intestinal epithelium with a mucus layer, which varies in terms of thickness, organization and composition. The small intestine is coated with a single thin, loose and penetrable layer, probably due to the rather sparse colonization and the antimicrobial peptides secreted from Paneth cells (8, 9). In the colon, the mucus consists of two layers, a loose outer layer and a dense inner layer attached to the epithelium (10). The inner layer serves as a physical barrier, while the outer layer provides a habitat and food source for certain commensal microbe populations. Due to this complexity, adequate reconstitution of the intestinal mucosa is crucial to maintain homeostasis of epithelial linings.

The increasing relevance of intestinal wound healing given and the significance of its dysregulation on patients (11), future therapeutic strategies will broaden the focus on aspects to improve mucosal repair. While this approach offers great potential, further research is necessary to enhance our knowledge about interactions between mucosal and luminal factors. In this review, we address the current roles of epithelial cells and microbiota in intestinal wound healing and discuss potential therapeutic strategies.

## Mucosal healing in the gut

Dysfunctional healing of the intestinal mucosa affects a large spectrum of patients resulting in a significant clinical relevance (12). Importantly, mucosal healing remains to be a double-edged sword since it has to be taken into account that persistent inflammation can result in uncontrolled overstimulation of proliferative pathways leading to the formation of neoplastic lesion and subsequent carcinogenesis. While reasons for the initial mucosal damage can be inflammation- or mechanical-related, mechanisms of wound repair are largely independent of the cause and consist of a multi-step process with various factors and cell types being involved (13). The complex process of mucosal healing, which needs to be clearly distinguished from the daily perpetual epithelial renewal driven by progenitor and stem cells, is well-regulated but dysregulation at any stage might result in insufficient wound closure with compromised gastrointestinal function or leakage. Importantly, maintaining adequate mucosal homeostasis consists of more than just closure of the epithelial lining since the intestinal mucosa is responsible to preserve barrier function but also to transport nutrients (14, 15). While factors such as environmental aspects or vascular insufficiency due to atherosclerosis, diabetes, aging or smoking are also relevant for wound healing in the gut, they are characterized by a different pathobiology and need to be addressed systemically. In contrast, factors which can be addressed locally and contribute to mucosal homeostasis and wound repair are epithelial cells and microbiota. Both sites are interacting closely and demonstrate a significant impact on the multi-step process of mucosal healing but despite that fact, they have no role in current treatment concepts. While clinicians and scientists mainly focused on interactions between microbiota and the local immune system in the past, recent studies have addressed the role of mucosa-associated microbiota with the relationship between epithelial cells and luminal bacteria being crucial for health and disease (16, 17).

## The role of epithelial cells and junctional proteins on intestinal wound healing

The coordination of the multi-step process of intestinal wound repair depends on the complex interplay between epithelial cells, immune cells and microbiota (18, 19). It consists of several overlapping stages which result in the re-establishment of the epithelial barrier through successful wound closure. After initial homeostasis, the inflammatory stage is driven by mucosal injury and mainly defined by the infiltration of neutrophils followed by macrophages and monocytes (20). In parallel and partially triggered by inflammation, epithelial cells remodel their cytoskeleton and start migrating and proliferating to achieve epithelial restitution. Finally, the restoration of mucosal homeostasis is completed by the differentiation of wound-associated epithelial cells (18).

Epithelial cells have an important role in segregating the intestinal microbiota from mucosal and submucosal linings but they are also participating in different pathways resulting in adequate mucosal healing. Various chemokines and growth factors are involved in the complex process of intestinal wound repair. For instance, small GTPases of the Rho family such as Rho and Rac contribute to the remodeling of the cytoskeleton with epidermal growth factor (EGF) and hepatocyte growth factor (HGF) and their signaling pathways leading to mucosal restitution and cell proliferation (21, 22). While the role of those factors and proteins is well established, recent studies have focused on the role of epithelial cells and their junctional proteins in regard to intestinal wound repair. In line with the relevance of GTPases mentioned before, Flemming et al. showed that loss of Desmocollin-2 (Dsc-2), a desmosomal cadherin, significantly delays epithelial cell migration in the gut due to the altered activity of GTPase Rap1 (23). While it remains to be confirmed that Dsc-2 controls Rap1 *via* Pkp3, the authors also demonstrated a functional interplay between Dsc-2 and integrin *β*1 and *β*4, thus, arguing for Dsc-2 as a key contributor in intestinal mucosal healing. In contrast, Desmoglein-2, another desmosomal cadherin which interacts closely with Dsc-2, is required for intestinal barrier integrity (24), but no effect on mucosal healing has been demonstrated to date, thus, underlining the complex interplays and functions of junctional proteins.

In line with the important role of epithelial cells and its junctions for intestinal wound repair, Reed et al. demonstrated that epithelial-expressed CD47 significantly effects mucosal healing *in vitro* and *in vivo* (25). While selective intestinal knockout of CD47 resulted in decreased mucosal healing following DSS colitis and biopsy wounding, it was shown that CD47 regulates mucosal repair again through a *β*1 integrin-dependent FAK-Src-p130Cas pathway. Similarly, other studies provide additional evidence for a direct effect of CD47 expression through that signaling pathway on intestinal wound closure. CD47 might be linked to FAK *via* TSP-1 and TGF-β1 but the mechanistic connection of CD47 to TSP-1 and TGF-β1 is currently missing and needs to be proven in the future (26, 27). Interestingly, while CD47 is a glycoprotein, the functional role of glycans located at the intestinal epithelium in general and its effect on wound repair in particular receives increasing attention in recent years. We could show that targeting of sialyl Lewis glycans located on Cd44v6 on the apical site of intestinal epithelial cells positively effect intestinal wound healing *in vitro* and *in vivo* (28). While sialyl Lewis glycans are highly upregulated during chronic inflammatory bowel diseases such as Crohn’s Disease and Ulcerative Colitis, antibody-mediated ligation of epithelial expressed sialyl Lewis glycans significantly enhances epithelial cell proliferation and migration by activating a signaling pathway downstream of CD44v6 including Src-FAK (28). In line with that, there is an increasing focus on glycolisation of cells such as intestinal epithelial cells and its functional aspect while the relevance of glycans in wound repair has been addressed in other studies as well (29–31).

Based on the evidence presented above, the role of junctional proteins connecting epithelial cells in the gut might have been understated in the past. Future studies will demonstrate if other proteins related to tight and adhesion junctions are not only contributing to epithelial barrier stabilization but also to signaling pathways resulting in adequate mucosal healing. Following that, it might not be surprising that wound repair and barrier function are closely related and should be addressed collectively. However and regardless of future studies, intestinal epithelial cells and its junctional proteins can already be seen as major players in mucosal healing in the gut.

## The role of microbiota on intestinal wound healing

Born sterile, the neonatal intestinal tract is soon colonized with commensal enteric bacteria. Although the temporal patterns of colonization and the formation of a complex and dynamic ecosystem are unique to each infant, the composition and functional capabilities of the microbiota resemble those of an adult at the age of around 2.5 years (32–34). The total number of commensal bacteria vary greatly between the different sections of the gastrointestinal tract and reach their peak in the ascending colon. About 2,100 species classified into 12 different phyla were identified in humans, but 90% of the species belong to one of the four following phyla: *Proteobacterio, Actinobacteria, Firmicutes and Bacteroidetes* (35). The symbiosis between microbiota and host is part of an ongoing evolutionary process that established a barrier function with a separation the colonized microbes from the systemic tissues on the one hand, but providing a gateway for a physiologically relevant cross-talk on the other hand.

In case of an intestinal wound due to physical trauma, infection or inflammatory conditions, the intestinal barrier is dysfunctional, changing the interplay of microbiota and systemic tissue. Depending on the composition of the microbiota prior or even immediately after wounding, healing might be promoted or disturbed. Commensal bacteria seem to promote the initial stage of epithelial restitution as studies in germ-free mice showed impaired rates of epithelial cell migration (36, 37). Cell migration is critically dependent upon the formation of focal adhesions (38), a link between the extracellular matrix and the cytoplasmic cytoskeleton of the migrating cell which is controlled by an enzyme called focal adhesion kinase. Several studies were able to show that enteric microbiota activate focal adhesion kinase, thereby enhance epithelial restitution and promote repair of mucosal wounds in a redox-dependent manner (39–42). The commensal microbiota also influences the development and training of the innate and adaptive immune system (43). This process is modulated by pattern recognition receptor (PPR) expressed on intestinal epithelial cells (44). They include Toll-like receptors (TLR) amongst others and recognize microbe-associated molecular patterns (MAMPs) from commensal microbiota (45). The TLR signaling regulates the production of antimicrobial peptides, which in turn are required to prevent microbial encroachments towards the intestinal mucosa and thereby preserve gut homeostasis (46). However, TLR was found to be expressed on intestinal stem cells as well, inhibiting cellular proliferation in the intestinal crypts by microbial ligand-mediated activation (47). Moreover, enterocyte-specific TLR4 activation *via* LPS resulted in an increase of intestinal stem cell apoptosis, following the pathogenic pathway of necrotizing enterocolitis. On the other hand, there are studies providing evidence, that the cytosolic bacterial sensor Nod2 stimulates stem cell survival of intestinal organoids upon activation by peptidoglycan motifs (48). These muramyl dipeptides (MDP) is common on all bacteria, but crypt resident bacteria have been identified (49) and the released MDP may have a protective effect on intestinal stem cells (48). The close crosstalk between microbiota and intestinal epithelial cells seems to affect proliferation of enterocytes and consecutively the repair of intestinal wound healing. However, the course for regenerative capacity of the intestinal epithelial might be set early in life and dependent on the microbial colonization. Germ-free born mice co-housed with specific-pathogen free mice during weaning, showed endured changes in gene expression, especially erythroid differentiation regulator-1 (Erdr1). It localizes to intestinal stem and transit amplifying cells, which differentiate into all epithelial lineages including Paneth cells, tuft cells, enteroendocrine cells, goblet cells, and enterocytes along crypt-villus axis. As a consequence, mice would show increased intestinal epithelial proliferation and regeneration in response to mucosal damage (50).

Infiltrating immune cells, such as macrophages, are important components of the intestinal wound healing. Microbial metabolites or cell wall components affect the polarization of macrophaghes to a M2 state in a mouse model of colitis, augmenting intestinal wound healing (51). In case of inflammation or wounding of the gut, transmigrating neutrophils accumulate in the injured mucosa, altering the physiological parameters of the local microenvironment, which is mostly due to a decrease in oxygen levels resulting from the formation of reactive oxygen species (52). In addition, the amount of mucins, as a relevant food supply to the microbiota, is shortened in mucosal wounds (53), thus, affecting local microbial composition and maybe to some extent individual wound healing. On the contrary, Wrzosek et al. reported an increase in goblet cell differentiation and mucus production when *Bacteroides thetaiotaomicron* and *Faecalibacterium prausnitzii*, two short-chain fatty acids-producing bacteria, were introduced to germ-free mice and colonized their guts (54). Microbial metabolites seem to have various effects on the architecture and functions of the intestinal barrier. Short-chain fatty acids were shown to enhance epithelial proliferation and differentiation and support the restauration of the epithelial barrier upon tissue damage (55, 56). In mice, *Bacteroides ovatus* alleviated lipopolysaccharide-induced inflammation (57). Furthermore, *Bacteroides ovatus* produces indole-3-acetic acid that most likely promotes IL-22 production by immune cells, yielding beneficial effects in a mice colitis model (58). In a mouse endoscope-wounding model, creating uniform lesion in the colonic mucosa of wild type mice, the abundance of anaerobic bacteria (*Akkermansia spp*.) increased substantially in early regenerative mucosa. In this study *Akkermansia muciniphilia* was applied intrarectally and mice showed superior wound closure and increased proliferation of enterocytes compared to mice that received inert control. However, this effect was dependent on the presence of the Fpr1 gene, which encodes for a necessary protein for respiratory burst in neutrophils (52).

However, despite the great potential of the microbiota and mucosal healing, another important aspect of microbiota-associated wound healing is the potential association between microbial composition and inflammation-associated carcinogenesis. Due to the great effects on cell proliferation and barrier integration, specific microbial species have also been demonstrated to facilitate the formation of pre-neoplastic lesions with one study showing a different microbial diversity in patients with IBD-related colorectal cancer. The importance is further underlined by different incidences between IBD-related colon and small bowel cancers which might be related to differences in microbial compositions. Therefore, further research is necessary to address this issue to evaluate the impact of the microbial diversity on the overall IBD-cancer prognosis as well as to identify the potential of probiotics to limit the overgrowth of pathogenic microbial species.

**Figure 1:**
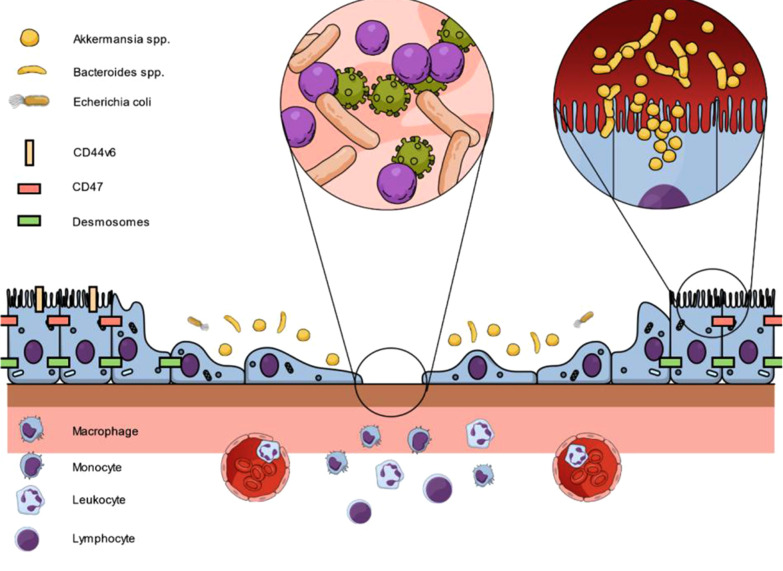
Interaction between epithelial cells, junctional proteins and microbiota on intestinal wound healing to restore mucosal homeostasis. Following epithelial injury, proteins such as CD44v6 or CD47 are upregulated at epithelial cells adjacent to the wound. In addition, luminal microbes are in close contact with intact epithelial cells. As a result, cell proliferation and migration is controlled and supported by the presented pathways to restore epithelial linings.

In a nutshell, there is consistently increasing evidence that intestinal wound healing is orchestrated by the microbial-epithelial interface (Figure 1). However, due to the inter-individual differences in the composition of the enteric microbiota, potential dysbiosis in commensal and pathogenic microbes and the circumstances under which the mucosal wounding occurs will challenge the results of this fundamental research in a bench-to-bedside translation. In addition, the enteric microbiota generates a vast variety of metabolites of largely unknown functions in the modulation of host cellular events.

## Future aspects

While the medical history teaches the pathophysiological misleading thesis of monomicrobial infections (59), the results on the fundamental research of the microbial-epithelial interface make it hard to believe that a single pathogenic microbe is causative for the underlying disease. While the mere presence of a pathogen will not cause the disease, it is a multifactorial disorder that leads to dysbiosis and ultimately the abundance of the microbe which meanwhile gained or activated virulence genes turning into a harmful aggressor for the host. In surgically created intestinal wounds, the use of antimicrobial or immunosuppressive drugs, extent and length of surgery, and early recovery pathways including nutritional aspects will have a relevant impact on the changes in enteric microbiota and thus on the healing of intestinal wounds. In endogenously developed intestinal wounds, e.g. due to inflammatory bowel disease, changes in microbiota might be a consequence of previous intestinal barrier breakdown as a function of dysfunctional intercellular adhesions or misguided inflammation. While there is data suggesting that Collagenase-producing and antibiotic-resistant organisms are more prevalent in anastomotic leak infections, precise adjustments in clinical management to prevent the local dysbiosis remain to be found (60). A promising approach seems to be a dietary change prior to surgery showing a preventive effect in developing anastomotic leakage after colostomy in mice (61). In addition, the transfer of living microorganisms, mostly Lactobacillus phyla, has exerted benefical effects on mucosal healing in murine models of DSS-colitis and gastric ulcera (62).

Besides addressing the microbiota, targeting proteins on intestinal epithelial cells such as desmosomes, CD47 or sialyl Lewis glycans relevant for epithelial migration and proliferation with novel medication has great potential as well and offers another target to improve intestinal mucosal healing (23–25, 28). To date, current research demonstrates great effects on wound healing but the promising results are limited to *in vivo* experiments, thus, translation to clinical studies is necessary to evaluate the disease-specific relevance in detail. Based on the postulated results, the innovative approach of targeting mucosal healing for therapy of IBD or to support anastomotic healing looks to be particularly promising. However, relevant questions such as how to obtain adequate local levels of the applicated substance by either oral or intravenous administration remain to be answered. In case of anastomotic healing, local application or injection could be an interesting approach which needs to be evaluated as well. Moreover, while for sialyl Lewis glycans antibody-targeting by GM35 to block shedding of the v6 domain from CD44v6 is necessary to support wound healing (28), for desmosomes and CD47 upregulation is aspired (23–25). Promising mechanisms for protein upregulation in humans are lacking, thus, more research is necessary to develop a realistic strategy for the latter proteins. In line with that, targeting and enhancing the involved pathways such as the *β*1 integrin-dependent FAK-Src-p130Cas pathway is an alternative which can be translated to clinical aspects more easily. Importantly, striving for a limited local effect of the administered agent is particularly relevant for CD47 since it is an ubiquitously expressed protein and a systemic impact of medical targeting of CD47 cannot be completely estimated upfront.

Finally, and most importantly, further development of treatment strategies does not mean to leave established aspects and concepts such as anti-inflammatory and immunosuppressive medication behind but to complement the current therapeutic regimen. Therefore, optimal disease-specific therapy should consider all aspect of the pathophysiology including epithelial cells, immune cells and microbiota in the future and will combine different targets to address mucosal healing better and to improve patient outcome.

## Conclusion

Recent research has demonstrated a major role of intestinal epithelial cells as well as microbiota on adequate mucosal healing in the gut with a close interaction between both sites. All targets involve the key intrinsic parameters of intestinal wound healing: The systemic condition of the patient, the mucosal cells, resident and transmigrating immune cells, and the enteric microbiota, both commensal and pathogenic (Figure 1). However, mucosal healing remains a double-edged sword since overstimulation of proliferative pathways can results in neoplastic lesions. Based on the postulated results, a repetitive re-evaluation of established principles for adequate wound repair in the gut is necessary to improve patient outcomes and disease control in the short- and long-term. To date, therapies of IBD and anastomotic healing mainly focus on immunosuppression and surgical aspects. However, the complex interplay not only of immune cells but also of junctional proteins and microbiota needs to be addressed in future studies and novel therapeutic protocols. Therefore, future investigators will need to consider all parameters in trying to piece this complex puzzle together. For clinicians, protection or restoration of the intestinal homeostasis should be the ultimate goal in the treatment of intestinal wounds.
